# Identification of genes involved in Ca^2+ ^ionophore A23187-mediated apoptosis and demonstration of a high susceptibility for transcriptional repression of cell cycle genes in B lymphoblasts from a patient with Scott syndrome

**DOI:** 10.1186/1471-2164-6-146

**Published:** 2005-10-21

**Authors:** Detlef Kozian, Valérie Proulle, Almut Nitsche, Marie Galitzine, Marie-Carmen Martinez, Beatrice Schumann, Dominique Meyer, Matthias Herrmann, Jean-Marie Freyssinet, Danièle Kerbiriou-Nabias

**Affiliations:** 1 Aventis Pharma Germany (Sanofi-Aventis group), Therapeutic Department Thrombosis and Angiogenesis, Industriepark Hoechst, Building H831, 65926 Frankfurt, Germany; 2 INSERM Unité 143, Hôpital de Bicêtre, 80 rue du Général Leclerc, 94276 Le Kremlin-Bicêtre, France; 3 Institut d'Hématologie et Immunologie, Faculté de Médecine, 4 rue Kirschleger, 67085 Strasbourg, France

## Abstract

**Background:**

In contrast to other agents able to induce apoptosis of cultured cells, Ca^2+ ^ionophore A23187 was shown to elicit direct activation of intracellular signal(s). The phenotype of the cells derived from patients having the hemorrhagic disease Scott syndrome, is associated with an abnormally high proportion of apoptotic cells, both in basal culture medium and upon addition of low ionophore concentrations in long-term cultures. These features are presumably related to the mutation also responsible for the defective procoagulant plasma membrane remodeling. We analyzed the specific transcriptional re-programming induced by A23187 to get insights into the effect of this agent on gene expression and a defective gene regulation in Scott cells.

**Results:**

The changes in gene expression upon 48 hours treatment with 200 nM A23187 were measured in Scott B lymphoblasts compared to B lymphoblasts derived from the patient's daughter or unrelated individuals using Affymetrix microarrays. In a similar manner in all of the B cell lines, results showed up-regulation of 55 genes, out of 12,000 represented sequences, involved in various pathways of the cell metabolism. In contrast, a group of 54 down-regulated genes, coding for histones and proteins involved in the cell cycle progression, was more significantly repressed in Scott B lymphoblasts than in the other cell lines. These data correlated with the alterations of the cell cycle phases in treated cells and suggested that the potent effect of A23187 in Scott B lymphoblasts may be the consequence of the underlying molecular defect.

**Conclusion:**

The data illustrate that the ionophore A23187 exerts its pro-apoptotic effect by promoting a complex pattern of genetic changes. These results also suggest that a subset of genes participating in various steps of the cell cycle progress can be transcriptionally regulated in a coordinated fashion. Furthermore, this research brings a new insight into the defect in cultured Scott B lymphoblasts, leading to hypothesize that a mutated gene plays a role not only in membrane remodeling but also in signal transduction pathway(s) leading to altered transcriptional regulation of cell cycle genes.

## Background

Several signaling pathways have been identified which promote the characteristic features of apoptotic cell death, including cell shrinkage, translocation of phosphatidylserine from the inner to the outer leaflet of the plasma membrane, internucleosomal DNA fragmentation and budding leading to disintegration into apoptotic bodies [1-3]. The biochemical and cellular mechanisms involved depend on the apoptogenic stimulus and may also be specific to the experimental model [4]. In contrast to other agents able to induce apoptosis, Ca^2+ ^ionophore A23187 elicits direct activation of intracellular signal(s). A23187 provokes caspase-independent apoptosis in Jurkat cells, which differs from Fas cross-linking by antibodies [3]. However, the apoptotic pathways induced by A23187, dependent on extracellular Ca^2+ ^ions [5,6], remain a matter of debate [7]. Although the effect of A23187 on gene transcription had been documented for various genes, there were no experimental data available on the overall pattern of gene expression in cells grown in the presence of this agent.

Scott syndrome is an extremely rare hereditary defect of swift egress (scrambling) of phosphatidylserine to the cell surface of stimulated platelets and blood cells, when challenged by stimuli resulting in rapid elevation of cytosolic Ca^2+ ^concentration [8-10], and the clinical phenotype is hemorrhagic [9]. Functional studies performed with B lymphocytes immortalized by Epstein-Barr virus (EBV)-transformation (B lymphoblasts) derived from three unrelated Scott syndrome patients demonstrated the same deficiency of rapid membrane response and suggested alteration in (a) Ca^2+^-dependent transduction pathway(s) [11-15]. Another characteristic of cultured Scott B lymphoblasts is a spontaneous tendency to apoptosis that is enhanced when A23187 is added to the culture medium [12,13,16,17]. We previously demonstrated using several methods that addition of 200 nM A23187 for 48 hours in the culture medium induced apoptosis in Scott B lymphoblasts more markedly than observed in control B cell lines [16]. It has been currently observed that cells originating from patients with genetic diseases or acquired pathologies may exhibit either spontaneous susceptibility or resistance to apoptosis, allowing the study of the role of the defective genes in the corresponding apoptotic processes [18-20]. The Scott B lymphoblasts thus appeared to constitute a unique model for the global investigation of changes in gene expression patterns during apoptosis and search of mutation(s) possibly accounting for the hemorrhagic phenotype associated with the syndrome.

In order to understand what controls the apoptogenic potential of the ionophore we examined by DNA microarray analysis whether A23187 provoked changes in the transcriptional patterns for 12,000 different mRNA sequences. We simultaneously checked if differential modulation of gene expression could account for the enhanced susceptibility of Scott B lymphoblasts to apoptosis as compared to B lymphoblasts derived from the patient's daughter or unrelated individuals. A change of expression of 109 genes was observed, including the coordinated repression of a set of 54 genes mostly involved in cell cycle progress. Our data illustrated that A23187-induced apoptosis correlates with transcriptional regulation of multiple genes and that global profiling analyses are important approaches for a better understanding of the involved mechanism(s). The results also showed a more pronounced gene repression in Scott B lymphoblasts, suggesting that the defect implies an element involved in (a) signal transduction pathway(s) promoting A23187-induced transcriptional regulation of cell cycle genes.

## Results

### Apoptotic characteristics of Scott B lymphoblasts

Spontaneous cell mortality of Scott B lymphoblasts *in vitro *has been previously shown [13,17] as well as their high susceptibility to 200 nM Ca^2+ ^ionophore A23187-induced apoptosis [16]. We confirmed using the cells cultured for the present study, the apoptogenic effect of the treatment for all B lymphoblasts (Table 1) and the much higher amount of apoptotic Scott B lymphoblasts in basal medium and upon addition of A23187.

**Table 1:** Effect of A23187 on apoptosis. The B lymphoblasts were cultured in basal (X-VIVO15) medium in the absence or presence of 200 nM A23187 for 48 hours. Percentages of FITC-annexinV positive cells were determined as described in Methods. Two Scott B lymphoblasts independently immortalized from the patient's lymphocytes, one daughter's and two control B lymphoblasts from unrelated individuals were used for these studies. Each cell line was analyzed twice. Results are means ± SD from the four experiments for Scott and control B lymphoblasts respectively and the two experiments for daughter's cells.

	**% of annexin V positive cells**	
	untreated	+ A23187
**Control**	18.5 ± 10.7	33.7 ± 17.6
**Daughter**	14.9 ± 8.6	29.6 ± 0.3
**Scott**	48.7 ± 7.6	64.7 ± 12.9

### Identification of 109 genes that are transcriptionally modulated in B lymphoblasts cultured in the presence of A23187

**Figure 1:**
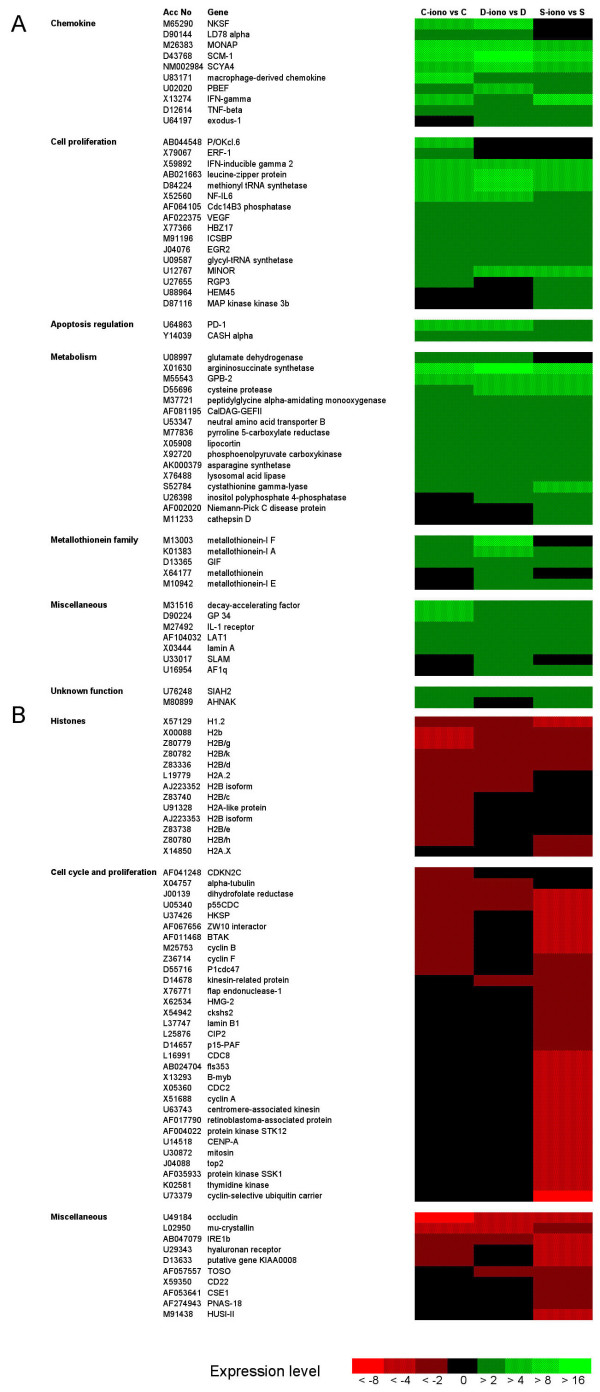
**Cluster analysis to compare the fold changes in gene expression in cells treated by A23187**. Cluster analysis allowed to directly compare the fold changes in gene expression for each one the three cell lines respectively (C: control B lymphoblasts; D: daughter's B lymphoblasts; S: Scott B lymphoblasts). Ratios determining the fold changes in gene expression due to treatment for 48 hours with 200 nM A23187 were examined for each one of the three cell types. The hierachical clustering of the selected genes (fold change of gene expression >2 with a P-value of <0.02 in at least one of the three cell types, see additional file 1) was performed. Without changing their respective hierarchical order, the genes were then grouped by functional category. Differential gene expression in A23187-treated versus non-treated cells is color coded as indicated at the bottom of the figure (fold-changes < 0.5 [see Additional file 1] correspond here to <-1/0.5, i.e. <-2). (A) Cluster analysis of up-regulated genes. (B) Cluster analysis of down-regulated genes. For selected genes represented on the U95Av2 GeneChips by more than one Probe Set ID [see Additional file 1], data corresponding to a single Probe Set ID were used for the cluster analysis and the gene lists in Tables 2 and 3.

The selection criteria for analyses of the hybridization data as defined in the Methods section enabled identification of 109 genes that were differentially expressed with a two-fold change or more of gene expression, after treatment of B lymphoblasts with 200 nM Ca^2+ ^ionophore A23187 for 48 hours [see Additional file 1]. Hierarchical clustering [21] (Fig. 1) and comparison of the fold change values (Tables 2 and 3), showed one group of 55 genes characterized by transcriptional activation (Fig. 1A, Table 2) and another group of 54 genes by repressed transcription (Fig. 1B, Table 3).

**Table 2:** Fold change of induced gene expression in B lymphoblasts treated with ionophore A23187. Genes listed here showed a 2-fold increase or more in gene expression in at least one of the three B lymphoblasts cultured for 48 h with 200 nM A23187. Data are fold changes calculated from hybridization values in treated versus non treated cells for each one of the B lymphoblasts respectively (see additional file 1). For each functional group, the genes were ordered by using a program for gene clustering. *Genes basally expressed differently in the cell lines (see additional file 2).

**Accession no.**	**control**	**daughter's**	**Scott**	**Gene definition**
				**Chemokines**
D90144	2.05	2.79	1.80	LD78 alpha
M26383	12.28	15.78	8.70	MONAP
D43768	11.58	30.28	11.87	SCM-1*
NM002984	5.46	8.43	4.63	SCYA4
U83171	9.70	2.44	2.29	macrophage-derived chemokine*
U02020	3.79	4.71	3.37	PBEF
X13274	4.19	3.20	9.17	IFN-gamma
D12614	2.83	3.91	3.01	TNF-beta
				**Cell proliferation**
AB044548	7.36	1.83	1.77	P/OKcl.6*
X79067	2.40	1.98	1.56	ERF-1
X59892	4.29	4.83	4.71	IFN-inducible gamma 2
AB021663	7.95	12.89	5.23	leucine-zipper protein
D84224	7.47	10.92	7.44	methionyl tRNA synthetase
X52560	4.32	4.10	3.70	NF-IL6
AF064105	3.44	3.00	2.67	Cdc14B3 phosphatase
AF022375	2.86	2.22	2.63	VEGF
X77366	2.66	2.51	2.42	HBZ17
M91196	2.02	3.70	3.47	DNA-binding protein
J04076	3.67	3.88	3.41	EGR2
U09587	2.01	2.13	2.20	glycyl-tRNA synthetase
U12767	3.84	4.00	7.02	MINOR
U27655	3.70	1.76	3.60	RGP3
U88964	-1.14	1.14	2.57	HEM45*
D87116	1.67	1.79	2.71	MAP kinase kinase 3b
				**Metabolism**
U08997	2.03	2.41	1.82	glutamate dehydrogenase
X01630	12.51	17.87	15.17	argininosuccinate synthetase
M55543	6.11	4.96	4.47	GPB-2*
M37721	2.20	2.27	2.77	peptidylglycine alpha-amidating monooxygenase
AF081195	2.48	3.98	2.18	CalDAG-GEFII
U53347	2.05	2.21	2.25	neutral amino acid transporter B
M77836	2.86	3.08	2.36	pyrroline 5-carboxylate reductase
X05908	2.18	3.45	2.77	Lipocortin*
X92720	2.05	2.10	2.34	phosphoenolpyruvate carboxykinase
AK000379	2.82	2.91	3.10	similar to asparagine synthetase
X76488	2.43	2.48	2.44	lysosomal acid lipase*
S52784	3.36	3.86	4.85	cystathionine gamma-lyase
U26398	1.98	3.69	2.77	inositol polyphosphate 4-phosphatase
AF002020	1.03	1.33	3.00	Niemann-Pick C disease protein
M11233	1.23	1.21	3.59	cathepsin D*
				**Metallothionein family**
M13003	2.85	10.83	1.90	metallothionein-I F*
K01383	3.62	4.60	3.90	metallothionein-I A
D13365	2.74	2.46	2.36	GIF
X64177	1.91	2.79	1.82	metallothionein
M10942	1.93	2.49	2.17	metallothionein-I E
				**Micellaneous**
M31516	5.63	4.00	3.57	decay-accelerating factor
D90224	4.84	3.93	2.16	GP 34*
M27492	2.28	3.60	3.07	IL-1 receptor*
AF104032	2.43	3.06	2.83	LAT1
X03444	2.79	2.02	3.66	lamin A*
U33017	1.90	2.62	1.56	SLAM
U16954	1.97	2.35	2.81	AF1q*
U64863	4.14	4.57	3.41	PD-1*
Y14039	2.25	3.48	2.51	CASH alpha*
U76248	2.47	2.81	2.68	SIAH2
M80899	2.13	1.33	2.69	AHNAK*

**Table 3:** Fold change of repressed gene expression in B lymphoblasts treated with ionophore A23187. Genes listed here showed a decreased gene expression (2-fold or more) in at least one of the three B lymphoblasts cultured for 48 h with 200 nM A23187. Data are fold changes calculated from hybridization values in treated versus non treated cells for each one of the B lymphoblasts respectively (see fold-changes < 0.5 in additional file 1 corresponding to <-1/0.5 in this table). For each functional group, the genes were ordered by using a program for gene clustering. *Fourteen genes are basally under-expressed in Scott B lymphoblasts compared to control cells.

**Accession no.**	**control**	**daughter's**	**Scott**	**Gene definition**
				**Histones**
X57129	-3.79	-3.59	-6.54	H1.2*
X00088	-6.80	-3.41	-3.62	H2b/r*
Z80779	-4.49	-2.11	-2.31	H2B/g*
Z80782	-2.96	-2.42	-3.41	H2B/k*
Z83336	-2.98	-2.12	-2.08	H2B/d*
L19779	-2.97	-2.01	-1.66	H2A.2*
AJ223352	-2.57	-1.71	-2.57	H2B/a*
Z83740	-2.92	-1.69	-1.83	H2B/c*
U91328	-2.81	-1.31	-1.65	H2A-like protein*
AJ223353	-3.12	-1.92	-1.27	H2B/b
Z83738	-3.08	-1.88	-1.98	H2B/e*
Z80780	-3.06	-1.84	-2.85	H2B/h*
X14850	-1.45	-1.40	-3.20	H2A.X
				**Cell cycle and proliferation**
AF041248	-2.58	-1.78	-1.84	CDKN2C*
X04757	-2.53	-2.55	-1.31	alpha-tubulin
J00139	-2.35	-2.35	-5.23	dihydrofolate reductase
U05340	-3.12	-2.44	-6.60	p55CDC
U37426	-2.07	-1.41	-5.10	HKSP
AF067656	-2.18	-1.26	-4.08	ZW10 interactor
AF011468	-2.20	-1.97	-6.05	BTAK
M25753	-2.13	-1.91	-7.30	cyclin B
Z36714	-2.20	-1.94	-3.74	cyclin F
D55716	-2.04	-1.57	-3.25	P1cdc47
D14678	-1.92	-2.05	-3.63	kinesin-related protein
X76771	-1.85	-1.17	-2.75	flap endonuclease-1
X62534	-1.38	-1.15	-3.20	HMG-2
X54942	-1.43	-1.48	-2.79	ckshs2
L37747	-1.65	-1.63	-2.84	lamin B1
L25876	-1.91	-1.69	-3.63	CIP2
D14657	-1.82	-1.27	-3.35	mRNA for KIAA0101 (p15-PAF)
L16991	-1.92	-1.78	-4.08	CDC8
AB024704	-1.76	-1.86	-4.60	fls353
X13293	-1.93	-1.69	-5.74	B-myb*
X05360	-1.84	-1.04	-4.12	CDC2
X51688	-1.62	1.05	-5.23	cyclin A
U63743	-2.00	-1.96	-6.59	centromere-associated kinesin
AF017790	-1.85	-1.55	-4.40	retinoblastoma-associated protein
AF004022	-1.92	-1.62	-5.11	protein kinase STK12
U14518	-2.00	-1.65	-5.02	CENP-A
U30872	-1.68	-1.49	-5.00	mitosin
J04088	-1.70	-1.22	-5.16	top2
AF035933	-1.80	-1.21	-4.54	protein kinase SSK1
K02581	-1.91	-1.63	-5.02	thymidine kinase*
U73379	-1.79	-1.66	-8.96	cyclin-selective ubiquitin carrier
				**Micellaneous**
U49184	-11.24	-6.64	-4.10	occludin
L02950	-5.26	-4.49	-3.85	mu-crystallin
AB047079	-2.20	-2.54	-4.21	IRE1b
U29343	-2.11	-1.58	-4.07	hyaluronan receptor
D13633	-2.21	-1.72	-6.35	putative gene KIAA0008
AF057557	-1.75	-3.43	-3.45	TOSO
X59350	-1.95	-1.06	-3.43	CD22
AF053641	-1.46	-1.45	-2.65	CSE1
AF274943	-1.20	-1.06	-2.45	PNAS-18
M91438	-1.18	-1.02	-3.41	HUSI-II

### Up-regulation of 55 genes

The activation of transcription for each one of the 55 up-regulated genes respectively appeared to be mostly of comparable amplitude in the three Scott, daughter's and control B lymphoblasts cell lines. These genes belong to various functional categories, coding for cytokines, transcription or growth factors and proteins of cell metabolism (Fig. 1A, Table 2). Hybridization ratios comparing the expression levels, in the absence of A23187, in Scott and daughter's relative to the control B lymphoblasts [see Additional file 2], suggested that in some cases, such as for expression of *SCM-1 *or *macrophage-derived chemokine*, different up-regulation values for a given gene reflected variable basal expression. The effect of the treatment (Table 2) then appeared to be more effective when the basal expression was low. Sixteen up-regulated genes were basally expressed differently [see Additional file 2] and the others were similarly expressed in the three cell lines before A23187 treatment.

### Down-regulation of 54 genes mostly coding for histones and proteins involved in the cell cycle

A significant fold change for repression of the 54 down-regulated genes mostly occurred in A23187-treated Scott B lymphoblasts when compared with control B lymphoblasts for which repression was of lower amplitude (Table 3). These genes mostly code for histones and for proteins involved in the progression of the cell cycle. Sets of genes coding for essential components of the molecular mechanisms involved in movements of organelles, microtubules or chromosomes and for proteins participating in the cell cycle at the level of DNA replication, repair and recombination or nucleotide synthesis were also down-regulated in A23187-treated cells. Fold change values found in daughter's cells were either intermediate when compared to Scott and control B lymphoblasts, or similar to control.

The comparison of the expression levels of these genes in Scott and daughter's relative to control B lymphoblasts in the absence of A23187 indicated a reduced basal expression of a subset of 14 genes in Scott B lymphoblasts [see Additional file 2]. These genes code for 11 histones and for CDKN2C, B-myb and thymidine kinase (marked by an asterisk in Table 3). The other down-regulated genes were similarly transcribed in the three cell lines in basal culture conditions.

### Independent verification of array findings

**Figure 2:**
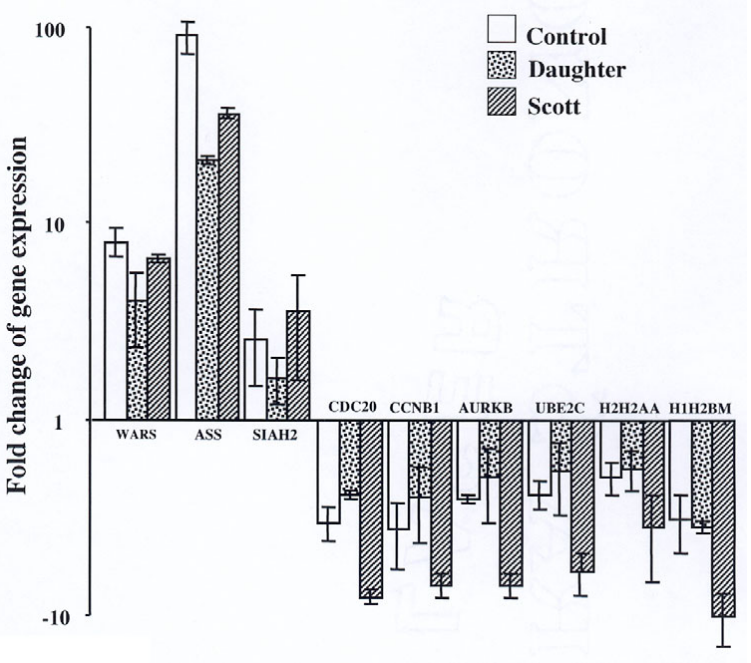
**Quantitative RT-PCR validation for a subset of genes differentially expressed by treatment with A23187**. RTs were performed with new RNA prepared from the treated and untreated cell lysates. For each PCR experiment, RT samples were analyzed at least in triplicate for the expression of a gene in parallel with *GAPDH *and 18S rRNA as described in Methods. Results are means ± SD from two independent PCR experiments with different RTs (each analyzed at least in triplicate) for the up-regulated genes and three independent PCR experiments for the down-regulated genes. The fold changes in expression level, i.e. 2^-ΔΔ*C*^T for the up-regulated genes calculated as described in Methods and -1/2^-ΔΔ*C*^T for the down-regulated genes (to compare with the values given in Table 3), are represented on a logarithmic scale. Some of the gene symbols listed in Table 2 and 3 have been recently renamed by Affymetrix [54] and the latest symbols were used for ordering the Assays-on-Demand. WARS: IFN-inducible gamma 2, Ac. N° X59892; ASS: argininosuccinate synthetase, Ac. N° X01630; SIAH2, Ac. N° U76248; CDC20: p55CDC, Ac. N° U05340; CCNB1: cyclin B, Ac. N° M25753; AURKB: protein kinase STK 12, Ac. N° AF004022; UBE2C: cyclin-selective ubiquitin carrier, Ac. N° U73379; H2H2AA: H2A.2, Ac. N° L19779; H1H2BM: H2B/e, Ac. N° Z83738.

To validate the microarray results, we measured the relative expression of an arbitrarily chosen subset of nine of the identified genes by real-time quantitative RT-PCR on new RNA preparations and RT (Fig. 2). Results confirmed the up-regulation of expression in the presence of A23187 of the genes coding for IFN-inducible gamma 2 (WARS), argininosuccinate synthetase (ASS) and SIAH2. The expression differences were of the same range values as those measured by microarrays analysis for WARS and SIAH2. Much higher values (for instance a fold change of 91 ± 17 for control B lymphoblasts compared to 12.5 from microarrays) were measured for ASS, demonstrating that for determining high difference levels the microarrays data saturate at lower values than those resulting from quantitative RT-PCR. Determination of mRNA levels using quantitative RT-PCR for six down-regulated genes confirmed the fold change values found in the microarrays analyses and demonstrated the stronger repression in Scott B lymphoblasts (Fig. 2). These data provide validation of the gene expression changes identified by microarrays.

### Treatment with A23187 alters cell cycle progression

**Figure 3:**
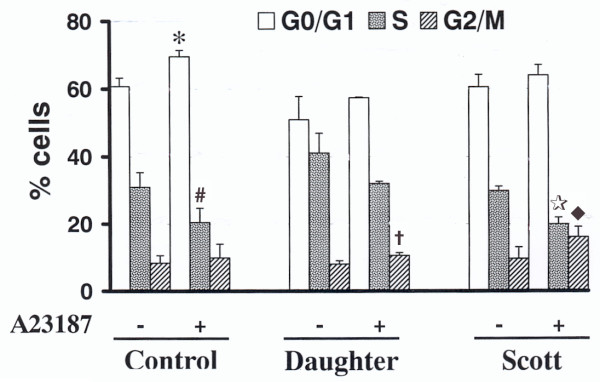
**Effect of A23187 on B lymphoblasts cell cycle**. The B lymphoblasts cultured in the absence or presence of 200 nM A23187 for 48 hours were stained with PI and analyzed for the proportion of cells in the different phases of the cycle by flow cytometry as described in Methods. Two independent experiments were performed with each one of five cell lines (two Scott B lymphoblasts independently immortalized from the patient's lymphocytes, one daughter's and two control cell lines from unrelated individuals). Data are means ± SD from the four independent experiments for Scott and control B lymphoblasts respectively and the two experiments for daughter's cells. For a given phase of the cell cycle and a cell type, *P *reflects the statistical differences between the percent of cells in the presence versus absence of A23187. * *P *= 0.0038, # *P *= 0.0016, † *P *= 0.0344, ★ *P *= 0.004,  ◆ *P *= 0.0134.

Cell cycle analyses were performed in order to check whether the decrease in expression of *histones *and cell cycle-related genes in A23187-treated cells correlated with changes in cell cycle progression. The cells continued to cycle in the presence of 200 nM A23187, although 10% decrease of populations in S phase was observed, suggesting partial blockade from G1 to S in all of the three cell lines (Fig. 3). However, cell cycle profiles for A23187-treated Scott, daughter's and control B lymphoblasts were differently affected. Scott cells exhibited a 2-fold increase of cells in G2/M and a very low enhancement of cells in G1. Conversely, with few changes in the number of cells in G2/M, the number of control B lymphoblasts in G1 was increased in the presence of A23187, coinciding with diminished proportion of cells in S phase. Intermediate values were found for daughter's B lymphoblasts. Therefore, it appears that treatment with A23187 modifies the cell cycle with, in Scott cells, a marked increase in G2/M populations in correlation with blocked progression to G1.

## Discussion

The spontaneous tendency to apoptosis is presumably a consequence of the still unknown mutation in Scott B lymphoblasts rather than due to EBV transformation. Other cases have been described, showing that EBV-transformed B cell lines, independently of viral transformation, retain the original characters of the B lymphocytes from which they originate [22]. Indeed, several B lymphoblast cell lines derived from the patient with Scott syndrome having provided the cells for this study as well as from the first described isolated case, all present a spontaneous tendency to apoptosis in culture [12,16,17]. Therefore, we expected that a better knowledge of the mechanisms underlying the spontaneous and A23187-induced apoptosis in Scott B lymphoblasts would be helpful for the identification of candidate gene(s) for the mutation(s).

Previous reports on the effects of A23187 on a variety of cultured cells pointed at apoptosis associated with induction of early response genes partly dependent only on caspase/Bcl-2 pathways [3,23,24]. However the signaling pathways controlling A23187-induced cell death are not known. Several studies have demonstrated that Ca^2+ ^ionophores are able to regulate gene transcription *in vitro *but the effects varied according to concentration, time course of the treatment and cell type [25-28]. In this study, although a possible effect of A23187 treatment for 48 h on the stability of the various mRNAs cannot be excluded, the fact that groups of genes were modulated in the same directions suggests transcriptional regulation. These genes could be, at least in part, regulated as downstream targets for early response genes coding for transcription factors or regulators.

Execution of apoptosis can be regulated by specific transcriptional factors and further modulated by cytokine-triggered signaling pathways [29]. The cytokines up-regulated in this study are markers of lymphocyte activation and mediators of inflammation [30-32] and may play a role in the modification of the B lymphoblasts leading to cell death. Additional genes up-regulated by A23187-treatment (see Table 2) code for proteins involved in cell proliferation [33], metabolism, protein synthesis, translation, tumor cell proliferation or transcription and may either promote cell death [23,34] or be markers of phenotypic changes. The up-regulated genes mostly appear to be similarly expressed and modulated in all of the three B cell lines and one could assume at least a partial role for these genes in the apoptotic characters of the treated cells although the importance of a specific gene cannot be deduced from this study. For instance, all the proteins coded by the three up-regulated genes analyzed to validate the microarrays data (WARS, ASS, SIAH2) were all demonstrated in other studies to be either involved in apoptosis or anti-proliferation [35,36] and/or markers of induction by interferon gamma [36,37].

Several mechanisms may simultaneously participate in the specific effects of A23187 on gene regulation, depending on the transcription machinery of individual genes. Our study further demonstrated that the treatment of Scott B lymphoblasts with A23187 provokes a down-regulation of a restricted set of 54 different genes. The transcription level of these genes was repressed more than two-fold in A23187-treated Scott B lymphoblasts and also decreased, although to lower extent, in daughter's and control B lymphoblasts.

Interestingly, at least 31 out of these 54 genes code for proteins involved in the formation of essential complexes during the onset of cell cycle or in cell cycle progress, and 13 of them code for histones. Genes expressing positive and negative regulators of the cell cycle were repressed, suggesting a complex effect of the treatment on cycle progression. Among the repressed genes are those coding for the cyclin-dependent serine-threonine kinase cdc2 and the subunit cyclins A and B, each being involved in the control of entry into mitosis and cell cycle advancement [38,39]. Remarkably, the gene encoding the cyclin-selective ubiquitin carrier, which participates in the destruction of cyclins A and B promoting exit from mitosis into G1 of the next cell cycle [40], is also repressed. Several down-regulated genes, coding for serine/threonine kinases (STKs in Table 3), are implicated in the segregation of chromosomes during mitotic cell division [41]. Other genes code for transcription factors or regulators, such as B-myb, which are important for chromosome segregation [42].

Decrease of *histones H1.2*, 3 *H2A *and 9 *H2B *expression is a characteristic feature observed in A23187-treated cells, including daughter's and control B lymphoblasts. Histones, of which genes are transcriptionally modulated as the cell cycle progresses, play key roles in the structural and transcriptional properties of the chromatin [43]. If the repressed genes are regulated at the transcriptional level in a coordinated fashion, one may hypothesize that common promoter elements may direct the observed pattern of regulation. Remarkably, expression of a large portion of the down-regulated genes is controlled by member(s) of the E2F family transcription factors. Among these genes are *histones, cyclins A *and *B*, *B-myb*, *Cdc2*, *Bub1*, *thymidine kinase*, *dihydrofolate reductase, top2*, *Flap endonuclease1 *[44]. E2F-DP1 heterodimers, essential for the G1/S phase transition bind to promoter elements and are negatively regulated by the hypo-phosphorylated retinoblastoma protein Rb [45]. A role for E2F in regulating gene expression of several *histones *subtypes correlates with the presence of consensus sequences for E2F binding in the shared *H2A/H2B *promoters [46]. However, the location of these sequences in the promoter domain is closer to the *H2A *side rather than to that of *H2B *(corresponding to most of the *histones *genes repressed in this study). This observation, together with the fact that only a restricted fraction of the known E2F target genes were repressed, suggests complex regulatory mechanisms for transcriptional regulation of genes down-regulated in the presence of A23187.

The data suggest a link between G0/G1 or G2/M cell cycle arrests and apoptosis. Similar relationships were previously demonstrated through various studies (see [47] and as shown for the effect of several anticancer agents [48-50]). Furthermore, the individual inhibition or knock-down of several genes repressed in the presence of A23187, such as *top2 *or *lamin B1*, was shown to induce apoptosis in HeLa cells [47,51,52]. Our results suggest that the down-regulation of cell cycle genes explains, at least partly, the apoptogenic effect of A23187. Although the analysis of the cell cycle phases did not show significant differences between the three B cell lines under basal conditions, A23187 treatment clearly changed the cell cycle profiles for all cell types. The presence of A23187 in culture medium diminished the transcription of genes important for progression of the cell cycle at the G1 phase correlating with an increase of cells in G1 and decrease of S phase cells. This was observed in all cell types, correlating with the down-regulation of cell cycle genes, although with lower change levels in daughter's cells and controls. Moreover, significant blockade of cells in G2/M phase was mainly observed for Scott and (at a lesser extent) daughter's B lymphoblasts.

Networks linking signaling and cell proliferation have now been widely described, connecting for instance Ras effector/extracellular signal-regulated kinase pathway with the Rb/E2F pathway, controlling either cell proliferation, differentiation, cell growth arrest or apoptosis [53]. These observations suggest that the expression pattern of the genes down-regulated by A23187 is associated with the apoptotic tendency of the cultured Scott B lymphoblasts. The present work leads to the hypothesis that a mutation in Scott cells would reside in a signaling pathway component shared by the membrane remodeling process and the transcriptional regulation of a subset of cell cycle genes upon addition of the Ca^2+ ^ionophore.

## Conclusion

The apoptogenic effect due to the addition to cultured cells of 200 nM A23187 for 48 hours correlates with an overall pattern of gene regulation involving at least a hundred genes and demonstrating the coordinated regulation of sets of them. Analysis of gene regulation upon treatment also represented a new approach to understand the still unexplained defective mechanism(s) in Scott cells. The results orientate the future work toward the exploration of a signaling defect in Scott B lymphoblasts upstream of the transcriptional machinery for genes participating in cell cycle progress and down-regulated with the treatment.

## Methods

### Cell lines

The cases of the propositus with Scott syndrome and of her daughter have been previously described and familial study has suggested a homozygous status for the propositus [13]. Control B lymphocytes were obtained from consenting and informed volunteers unrelated to the patient. All B lymphoblasts used in this study were B lymphocytes transformed in our laboratory by EBV-infection into proliferating cell lines as already reported [13]. The Scott phenotype, *i.e. *a lack of exposure of procoagulant phosphatidylserine due to defective membrane remodeling, was constantly observed in independently EBV-infected B cells from the propositus [13]. The B lymphoblasts derived from the propositus' daughter (daughter's B lymphoblasts in this study) exhibited *in vitro *functional properties coinciding with heterozygous status, although this individual denied any hemorrhagic tendency [13]. B lymphoblasts were routinely seeded at 2 × 10^5 ^cells/ml and expanded to 6 × 10^5 ^cells/ml in X-VIVO15 culture medium (BioWhittaker, Cambrex Bio Science, Verviers, Belgium, [Ca^2+^]_free _= 1.8 mM), without any other additive.

### Culture conditions in the presence of Ca^2+ ^ionophore A23187

The B lymphoblasts (8 × 10^7 ^cells) were seeded at 5 × 10^5 ^cells/ml in X-VIVO15 medium. To treat the cells, A23187 (200 nM final concentration, Calbiochem, La Jolla, CA) was added to the cultures four hours later. Treated and non-treated cells were further cultured for the indicated times before harvesting.

### Assessment of apoptosis

Detection and quantification of apoptosis were performed on 6 × 10^5 ^cells, washed twice with Hanks' balanced salt solution (Sigma-Aldrich), and diluted in 300 μl of the same solution supplemented with 1 mM CaCl_2_. Fluorescein isothiocyanate (FITC)-Annexin V solution (BD Biosciences, Pharmingen) was added to 5% vol/vol. The cells were then incubated for 20 min at room temperature before analysis to quantify the apoptotic cells that expose PS with the CELL Quest software using a FACSscan flow cytometer (BD Biosciences).

### Cell cycle analysis

For cell cycle studies, 3 × 10^6 ^cells were collected and incubated in the dark at 4°C for 20 min in 500 μl of phosphate buffer saline supplemented with 0.1% Triton X-100, 0.5 mg/ml RNase A (type I, Sigma) and 50 μg/ml propidium iodide (PI, CN Biosciences, Nottingham, UK). Data acquisition were performed by flow cytometry analysis by gating on an appropriate area to exclude cell debris and aggregate with the CELLQuest software using a FACSscan flow cytometer (BD Biosciences, San Jose, CA, USA). Quantification of cells in the different phases of the cycle was then performed using the ModFit LT™ software (Verity Software House Inc., Topsham, ME, USA). Results are expressed as percentage of cells in each phase of the cell cycle.

### RNA extraction, cRNA preparation and microarray hybridization

B lymphoblasts were cultured for 48 h in the presence or absence of 200 nM A23187. Total RNA from cultured cells was isolated for double-stranded cDNA synthesis using Trizol™ reagent (Invitrogen Ltd, Paisley, UK) according to the manufacturer's instructions. RNA was further purified with RNeasy columns (Qiagen, Hilden, Germany). Ten μg of total RNA were subjected to first strand cDNA synthesis reaction using an oligo(dT)-primer with a T7-promoter sequence added to the 5'-end (Superscript Choice System, Invitrogen Ltd). After second-strand synthesis, double-stranded cDNA was purified by phenol/chloroform extraction, precipitated and diluted in nuclease-free water. Biotin-labeled cRNA was made by *in vitro *transcription using ENZO Bioarray High Yield Transcription kit (Affymetrix Inc, Santa Clara, CA, USA). The resulting cRNA was fragmented at 94°C for 35 minutes in 40 mM Tris-acetate buffer, pH 8.1, 100 mM K-acetate and 30 mM Mg-acetate. Human genome U95Av2 GeneChip^® ^microarray (see the updated list in [54]) was used to analyze gene expression patterns. For hybridization with GeneChip^®^, 12 μg of fragmented cRNA was incubated with 50 pM control oligonucleotide B2 (Affymetrix Inc.), 1X eukaryotic hybridization control (Affymetrix Inc.), 0.1 mg/ml herring sperm DNA and 0.5 mg/ml acetylated BSA and 1X hybridization buffer according to the manufacturer's instructions for 16 to 18 h at 45°C. Washing and staining were performed in Affymetrix GeneChip^® ^Fluidics Station using Affymetrix antibody staining and washing protocol. GeneChip^® ^microarrays were scanned with Agilent Gene Array (Affymetrix Inc.) scanner (100% PMT settings). Results for a given sample originate from two independent sample preparations, in vitro transcriptions and hybridization reactions performed in order to avoid any bias due to variations in sample treatment.

### Analysis of hybridization data

Scanned images were analyzed using Microarray Suite 4.0 (Affymetrix Inc.), which assigns an intensity that is a measure of the corresponding transcript abundance. Replicates were combined by computing the median of the replicate intensity. For each Probe Set ID, the expression ratios were obtained using both intensity and noise data through the PFOLD algorithm [55]. This provides an estimate of the expression ratio and also a P-value, which quantifies its statistical significance. All statistics and graphics related to Affymetrix analysis were performed using the GECKO software [21]. Ratios determining the fold changes in gene expression due to A23187 treatment were determined for each cell line, *ie*: Scott, daughter's and control B lymphoblasts. All further analyses were performed with genes displaying a 2-fold change or more in expression level and a P-value <0.02 for at least one of the three cell lines (see additional file 1). Data are deposited in NCBI's GEO (Gene Expression Omnibus site) [56] with accession N° GSE1028.

### Quantitative RT-PCR

Reverse transcription (RT) followed by quantitative real-time polymerase chain reaction (quantitative RT-PCR) was performed on new RNA preparations of the B lymphoblasts lysates to validate the microarray data. RT was performed with 4.5 μg of total RNA (100 μl final reaction) using the High-Capacity cDNA Archive Kit (Applied Biosystems, Foster City, CA). Quantitative PCR was performed using an ABI Prism 7000 Sequence Detector (Applied Biosystems). PCR was performed with 5 μl of 10 times diluted RT samples mixed in 96-well optical reaction plates with 20 μl of a solution containing the TaqMan Universal PCR master mix and a TaqMan Assay-on-Demand (Applied Biosystems) according to the manufacturer's instructions. The TaqMan Assays-on-Demand include the primers and a fluorescent TaqMan (6-FAM dye-labeled) probe allowing the specific amplification of a cDNA and its quantification via the determination of the threshold cycle (*C*_T_) value. The thermal cycling conditions were: 50°C for 2 min and 95° for 10 min, followed by 40 cycles of 95° for 15 s and 60° for 1 min. For a PCR experiment, each RT sample was analyzed at least in triplicate for every analyzed cDNA. Glyceraldehyde-3-phosphate dehydrogenase (GAPDH, for which gene expression was not regulated by A23187 treatment) and 18S rRNA were amplified from the same RT in separate reactions to normalize the results. The PCR experiments were repeated three times for the genes down-regulated by treatment with A23187 and twice for the up-regulated genes. Relative quantification of gene expression was carried out using the 2^-ΔΔ*C*^T method . The average (*C*_T_) value for GAPDH or 18S was subtracted from the average (*C*_T_) value for the analyzed cDNA (Δ*C*_T_). Difference in (Δ*C*_T_) values between A23187 treated and untreated samples (-ΔΔ*C*T) were used to calculate the relative change in gene expression ( = 2^-ΔΔ*C*^T).

## Authors' contributions

DK coordinated the hybridization experiments, collected the statistical data and contributed to initiate and design the study and to draft the manuscript. VP performed the analyses by flow cytometry to determine apoptosis and cell cycle progression. AN carried out the statistical analyses of the hybridization values. MG and MCM made preliminary experiments having originated the design of the study. BS conducted the microarray hybridizations. DM, MH and JMF were crucial to initiate and support the study and participated in its design and coordination. DKN contributed to initiate, design, and coordinate the study, carried out the cultures, preparation of cell extracts and quantitative RT-PCR, analyzed the significance of the genes regulations and drafted the manuscript. All authors participated in the final drafting of the manuscript. DK and VP contributed equally to the work.

## Supplementary Material

Additional File 1 **Excel file containing the 109 genes displaying in at least one of the three cell lines a two fold change or more in expression level due to A23187 treatment **. The list shows the Affymetrix Probe Set IDs, GenBank accession numbers, GenBank definitions, fold changes and p values. Results are sorted by GenBank definition, allowing the assemblage in the list of the different Probe Set IDs corresponding to a single gene. Click here for file

Additional File 2 Relative basal gene expression of the genes up-regulated by A23187 treatment (Table A) or down-regulated by A23187 treatment (Table B), in Scott B lymphoblasts or daughter's B lymphoblasts versus control B lymphoblasts. Click here for file
