# Supplementary material for: Identification of genes involved in Ca2+ ionophore A23187-mediated apoptosis and demonstration of a high susceptibility for transcriptional repression of cell cycle genes in B lymphoblasts from a patient with Scott syndrome
Source: BMC Genomics. 2005 Oct 21;6:146. doi: 10.1186/1471-2164-6-146 (PMC1312317; doi:10.1186/1471-2164-6-146)
Supplement: Additional File 2 — Relative basal gene expression of the genes up-regulated by A23187 treatment (Table A) or down-regulated by A23187 treatment (Table B), in Scott B lymphoblasts or daughter's B lymphoblasts versus control B lymphoblasts. [file 1471-2164-6-146-S2.doc]

Relative basal gene expression in Scott B lymphoblasts or daughter's B lymphoblasts versus control B lymphoblasts

|  |  |  |  |  |
| --- | --- | --- | --- | --- |
| A) Genes up-regulated by A23187 treatment | | | |  |
| Accession N° | D/C | S/C | Gene definition |  |
| M27492 | 3.13 | 6.68 | IL-1 receptor | |
| D90224 | 4.75 | 14.24 | GP34 | |
| X05908 | 4.54 | 15.78 | lipocortin | |
| U83171 | 4.61 | 8.78 | macrophage-derived chemokine | |
| X03444 | 2.11 | 2.78 | lamin A | |
| M80899 | 2.66 | 2.20 | AHNAK | |
| AB044548 | 4.43 | 2.41 | P/OKcl.6 | |
| U16954 | -1.03 | 2.29 | AF1q | |
| D43768 | -2.35 | 3.66 | SCM-1 | |
| X76488 | 1.33 | 2.80 | lysosomal acid lipase | |
| U64863 | 1.47 | 2.10 | PD-1 | |
| M11233 | 1.11 | -2.24 | cathepsin D | |
| M55543 | -4.05 | -1.55 | GBP-2 | |
| U88964 | -2.91 | -4.00 | HEM45 | |
| Y14039 | -2.48 | 1.11 | CASH alpha | |
| M13003 | -4.94 | 1.28 | metallothionein-I F | |

B) Genes down-regulated by A23187 treatment

| M91438 | -1.14 | 11.77 | *HUSI-II ^a^* |
| --- | --- | --- | --- |
| X13293 | -1.08 | -2.06 | *B-myb* |
| K02581 | -1.32 | -2.67 | *thymidine kinase* |
| X57129 | -3.48 | -5.06 | *H1.2* |
| AJ223352 | -3.24 | -2.30 | *H2B/a* |
| Z80780 | -3.14 | -2.37 | *H2B/h* |
| AF041248 | -2.99 | -4.03 | *CDKN2C* |
| Z80782 | -3.14 | -2.37 | *H2B/k* |
| L19779 | -3.52 | -4.45 | *H2A.2* |
| Z80779 | -4.05 | -2.61 | *H2B/g* |
| X00088 | -6.57 | -4.88 | *H2B/r* |
| Z83740 | -3.54 | -2.91 | *H2B/c* |
| Z83738 | -3.76 | -3.00 | *H2B/e* |
| Z83336 | -3.72 | -2.75 | *H2B/d* |
| AJ223353 | -4.70 | -1.95 | *H2B/b* |
| U91328 | -3.54 | -2.57 | *H2A-like protein* |

The basal gene expression levels in Scott B lymphoblasts or daughter's B lymphoblasts were determined relative to those in control cells for all of the 109 genes regulated by treatment with A23187 (D/C: daughter's B lymphocytes versus control B lymphocytes; S/C: Scott B lymphocytes versus control B lymphocytes). Genes exhibiting a ratio with a 2-fold change for at least one ratio (D/C or S/C) were ordered by gene clustering. A: genes up-regulated by A23187 treatment (listed in Table 2); B: genes repressed by A23187 treatment (listed in Table 3). *^a^*The acrosin inhibitor HUSI-II is expressed in Scott and not in the other B lymphoblasts. The other regulated genes listed in Tables 2 and 3 appeared to be similarly expressed in the three cell lines before A23187 treatment with ratios D/C and S/C between 0.5 (ie –2) and 2.
